# Determination of pain intensity, pain-related disability, anxiety, depression, and perceived stress in Polish adults with temporomandibular disorders: A prospective cohort study

**DOI:** 10.3389/fnint.2022.1026781

**Published:** 2022-11-02

**Authors:** Mieszko Wieckiewicz, Andrej Jenca, Piotr Seweryn, Sylwia Orzeszek, Adriana Petrasova, Natalia Grychowska, Orit Winocur-Arias, Alona Emodi-Perlman, Krzysztof Kujawa

**Affiliations:** ^1^Department of Experimental Dentistry, Wroclaw Medical University, Wroclaw, Poland; ^2^Clinic of Stomatology and Maxillofacial Surgery, Faculty of Medicine, University Pavol Josef Šafárik and Akademia Košice, Košice, Slovakia; ^3^Department of Dental Prosthetics, Wroclaw Medical University, Wroclaw, Poland; ^4^Department of Oral Pathology and Oral Medicine, The Maurice and Gabriela Goldschleger School of Dental Medicine, Tel Aviv University, Tel Aviv, Israel; ^5^Department of Oral Rehabilitation, The Maurice and Gabriela Goldschleger School of Dental Medicine, Tel Aviv University, Tel Aviv, Israel; ^6^Statistical Analysis Center, Wroclaw Medical University, Wroclaw, Poland

**Keywords:** temporomandibular disorders, pain, anxiety, depression, perceived stress

## Abstract

**Background:**

There is a need to assess a relationship between the psychoemotional state of patients and the occurrence and the intensity of pain and temporomandibular disorders (TMD) in relation to a Polish population. There are no such precision data in the literature relating to the population of big Eastern European country. The study conducted by the authors refer to a large group of male and female adult patients of the Polish population in a different age profile. As a result, this study provides a picture of the situation that also takes into account population characteristics that may affect the clinical situation of patients.

**Objective:**

The aim of the study was to assess the pain intensity, pain-related disability, anxiety, depression, and perceived stress among Polish adults with TMD as well as the association between psychosocial impairment and TMD.

**Materials and methods:**

This prospective cohort study included 219 adult patients from the Outpatient Clinic for Temporomandibular Disorders at the Academic Dental Polyclinic in Wroclaw. The patients completed validated questionnaires, and received a TMD diagnosis based on a standardized examination (Diagnostic Criteria for Temporomandibular Disorders) performed by a qualified dentist. A statistical analysis was carried out on the collected data.

**Results:**

Myalgia was the most commonly diagnosed condition among the TMD patients. Furthermore, the prevalence of perceived stress, anxiety, and depression was high in the studied sample. Females showed more depression symptoms than males, while the level of anxiety and stress was similar in both groups. The most statistically significant correlation was observed between the group of masticatory muscle disorders and the level of depression, stress, pain intensity, and pain-related disability.

**Conclusion:**

Due to the high prevalence of increased levels of anxiety, depression, and perceived stress, it is essential to screen the psychosocial status of Polish TMD adult patients. Psychosocial status may have an impact on a studied patient’s response to treatment and pain intensity, and pain-related disability. Therefore, Polish TMD adult patients should be provided with management based on an interdisciplinary approach.

**Clinical trial registration:**

[https://clinicaltrials.gov/], identifier [NCT05183503].

## Introduction

Temporomandibular disorders (TMD) is a broad term that refers to a heterogenic group of conditions affecting the masticatory muscles, temporomandibular joints, and the surrounding hard and soft structures (Durham et al., 2015). TMD are considered as a global problem and are characterized by orofacial pain, joint sounds such as clicking or crepitus, and limitations in jaw movements (Durham et al., 2015). TMD are the second major cause of orofacial pain as well as the second main cause of pain and disability in the musculoskeletal system after chronic low-back pain (Schiffman et al., 2014; Meloto et al., 2019). TMD also decrease the quality of life of patients, potentially limit their daily activities due to pain intensity, pain-related disability, and increase anxiety and depression. Moreover, they cause general health problems, decrease work productivity, and increase absenteeism (Peres et al., 2019).

The etiology of TMD is complex, multifactorial, and often unclear (Osiewicz et al., 2017). The biopsychosocial model encompasses a wide range of diseases and factors that may contribute to TMD. It integrates biological elements (structural disorders and functional disturbances) with psychosocial components (emotions, cognition, behaviors, reaction to stress and pain in the context of family, workplace, and community) (Schiffman et al., 2014; Durham et al., 2015).

TMD affects between 5 and 12% of the general population (Liu and Steinkeler, 2013). About 26–30% of young adults have at least one TMD symptom (Loster et al., 2017; Lövgren et al., 2018). The prevalence of TMD in adults over 45 years old is estimated at 2–7% and in those over 65 years old at 3–5% (Yadav et al., 2018). The prevalence of TMD among adults in Poland is 55.9% (Wieckiewicz et al., 2020). It is commonly assumed that women are more susceptible to TMD than men (Wieckiewicz et al., 2014).

Many studies have indicated a strong association between TMD and psychosocial symptoms including depression, somatization, and anxiety (Manfredini et al., 2009, 2011; Ohrbach et al., 2010). When compared to pain-free controls, patients with chronic pain conditions have been shown to have high levels of psychosocial impairments (Canales et al., 2019). According to some researchers, psychosocial factors limit the response of TMD patients to conservative treatment and may increase their risk of developing chronic TMD (Bonjardim et al., 2005; Monteiro et al., 2011; Litt and Porto, 2013; Huttunen et al., 2018). Therefore, it is necessary to assess TMD patients for various types of psychosocial disorders, in order to make appropriate clinical decisions and initiate proper management.

Pain-related TMD are closely related to social and psychological factors which was confirmed during the resting-state functional magnetic resonance imaging (RS-fMRI) of spontaneous brain activity. Numerous studies point to the relationship between spontaneous brain activity and its related functional connectivity (FC) within the mood-regulating circuits (MRC) and emotional symptoms pain-related TMD patients. In the literature we can find also research with the use of magnetic voxel-based morphometry (VBM). Findings show that TMD, like other chronic pain states are associated with changes in brain morphology (Gerstner et al., 2011; Suenaga et al., 2016; Barkhordarian et al., 2020; Budd et al., 2022; Chen et al., 2022).

The available literature data on the association between TMD and psychosocial impairment may aid in the diagnosis and management of TMD. Unfortunately, the prevalence and epidemiology of pain intensity and pain-related disability, depression, anxiety, and perceived stress in Polish TMD patients are unknown. As a result, the existing data cannot be applied to this group, and hence there is an epidemiological and information gap in this area for a large European country.

The objectives of the present study are to assess the pain intensity, pain-related disability, anxiety, depression, and perceived stress among Polish adults with TMD, as well as the association between these psychosocial symptoms and TMD, and to use the obtained data for appropriate diagnosis and management of TMD.

## Materials and methods

### Participants and study design

This prospective cohort study included 219 Polish adult patients who were receiving treatment in the Outpatient Clinic for Temporomandibular Disorders at the Academic Dental Polyclinic in Wroclaw, Poland.

All the included patients provided signed consent to use their data for research purposes. The study was conducted in accordance with the principles of the Helsinki Declaration, and the protocol was approved by the Bioethical Committee of the Wroclaw Medical University (No. KB-165/2021).

The study has been retrospectively registered in database of clinical studies (ClinicalTrials.gov) on 6th January 2022 and received the following registration number NCT05183503.

### Inclusion and exclusion criteria

The inclusion criteria for the study were as follows: (1) age of 18 years or above and (2) diagnosis of TMD based on the DC/TMD (Diagnostic Criteria for Temporomandibular Disorders) examination (Schiffman et al., 2014). The exclusion criteria were as follows: (1) severe neurological and/or mental disease, (2) use of medications that can significantly disturb the neuromuscular function and/or logical contact, (3) alcohol and/or drug addiction, (4) diagnosis of active cancer, and (5) pregnancy.

### Data collection

Data were collected from the patients between November 2018 and December 2020. First, the patients were asked to complete questionnaires, and then a clinical examination of masticatory muscles and temporomandibular joints was performed by a qualified dentist based on the DC/TMD protocol (Schiffman et al., 2014).

### Questionnaires (diagnostic criteria for temporomandibular disorders axis II)

#### Graded chronic pain scale

The graded chronic pain scale (GCPS) is part of Axis II screeners of DC/TMD protocol (Schiffman et al., 2014). It includes six questions regarding facial pain in the last 3–6 months, which are evaluated on a scale from 0 to 10. A score of 50/100 or above indicates the high intensity of pain. The seventh question refers to the number of days that the patient has been unable to engage in usual activities due to facial pain. The final score is calculated from three subscales (characteristic pain intensity score, disability score, disability points score) and classifies patients into one of five pain severity grades (Von Korf et al., 2011). Grade 0 stands for no pain, grade 1 for low disability and low intensity, grade 2 for low disability but high intensity, grade 3 for high disability and moderately limiting, and grade 4 for high disability and severely limiting (Manfredini et al., 2010).

#### Generalized anxiety disorder scale-7

The generalized anxiety disorder scale-7 (GAD-7) is a seven-item questionnaire, which is a valid and reliable tool for screening anxiety (Löwe et al., 2008; Simoen et al., 2020). The questions concern the frequency of anxiety signs, worry, ability to relax, irritability, and related features (Löwe et al., 2008). Participants must choose one of the four options: “not at all,” “several days,” “more than half the days,” and “nearly every day.” Each answer is assigned a score of 0, 1, 2, or 3, respectively. A score of 5 or higher indicates mild anxiety, 10 or higher indicates moderate anxiety, and 15 or higher indicates severe anxiety. The sensitivity of this questionnaire has been estimated as 89% and specificity as 82% (Spitzer and Kroenke, 2006).

#### Patient health questionnaire-9

The patient health questionnaire-9 (PHQ-9) is another tool used in Axis II of the DC/TMD protocol (Von Korf et al., 2011). It is a validated, self-rating questionnaire for assessing depression symptoms (Spritzer et al., 1999; Kroenke et al., 2001). The questionnaire includes nine questions regarding patient wellbeing. Each item in the questionnaire is associated with a diagnostic criterion for major depressive episodes. The maximum possible score is 27, and the cutoff scores for mild, moderate, moderately severe, and severe depression are 5, 10, 15, and 20, respectively. The questionnaire has an overall accuracy of 85%, sensitivity of 75%, and specificity of 90% (Spritzer et al., 1999).

#### Perceived stress scale-10

The perceived stress scale-10 (PSS-10) is used to assess the intensity of stress caused by life situations during the last month (Taylor, 2015; Nielsen et al., 2016). It consists of 10 questions regarding various subjective feelings about problems, behaviors, and coping methods. The questionnaire is divided into two parts: part 1 examines adaptation symptoms and part 2 examines the coping ability. The patients have to choose one of the five options, but for some questions the scoring is inverted as follows: 0–4 points, 1–3 points, 3–1 point, and 4–0 points. The maximum possible score is 40, and scores 1–13, 14–26, and 27–40 indicate low stress, moderate stress, and high perceived stress, respectively.

### Clinical examination (diagnostic criteria for temporomandibular disorders axis I)

After completing the questionnaires, all the participants were subjected to a complete clinical examination. All the examiners who assessed the patients were qualified and experienced dentists (minimum 5 years of practice). They were trained and calibrated according to the protocol available on the official website of the International Network for Orofacial Pain and Related Disorders Methodology^1^ by a clinician who had 10 years of experience in TMD and orofacial pain management and was familiar with the DC/TMD examination protocol. It was ensured that the examiners had a solid understanding of English and professional terminology in English, as the study employed the original DC/TMD examination form (international version, 12 May 2013^2^). In addition, each examiner had access to the official DC/TMD examination instructional video in English.^3^ Diagnoses for patients were given for each side separately, and each side could have multiple diagnoses (Peck et al., 2014; Schiffman et al., 2014).

## Statistical analyses

Variables in statistical analysis: GCPC–nominal ordinal variable and PHQ-9, PSS-10, and GAD-7 are continuous variables. To analyze the relationship between PHQ-9, PSS-10, and GAD-7, they were transformed using the Box-Cox function and the Pearson correlation was used. On the other hand, to link the GCPC scale with other scales, a regression model was used, where the variable being explained is a categorical variable ordered (GCPC), and the explanatory variables are the remaining scales (PHQ-9, PSS-10, GAD-7), the ordered logistic regression was used (Venables and Ripley, 2002).

To analyze the relationship between GAD-7, PHQ-9, PSS-10, and TMD, age and gender the ordered logistic regression was used—separately for each of the analyzed scales. For controlling the I-type error when multiple testing is performed, the Bonferroni’s correction for the *p*-value was applied (*p*-value was multiplicated by the number of analyses run). The ordered logistic regression was performed with the use R-package “MASS” (Venables and Ripley, 2002).

## Results

### Sample characteristics

A total of 219 Caucasian Polish adult patients with TMD participated in the study. The sample included 163 women (74%) and 56 men (26%). The mean age of the patients was 40.06 (± 16.37), and the age range was 18–80. Females were in majority in the sample (3:1 ratio) and were statistically significantly older than men [women: mean age 42 years (±16.95), range 18–80; men: mean age 34 years (± 13.17), range 18–73] (Table 1).

**TABLE 1:** Age distribution in the study group.

		*N*	Mean	Min	Max	SD^†^
Study group	Age	219	40.05936	18.00000	80.00000	16.36709
Women group	Age	163	40.05936	18.00000	80.00000	16.94579
Men group	Age	56	34.48214	18.00000	73.00000	13.16882

^†^Standard deviation.

The distribution of patients in particular age groups was as follows: 18–35 years, *n* = 103; 36–55 years, *n* = 46; and >56 years, *n* = 70. No statistically significant correlation was observed between the DC/TMD diagnosis and the age of patients (*p* > 0.05).

The patients diagnosed with DC/TMD were classified into three groups: muscle pain (*n* = 159), joint pain (*n* = 120), and comorbid muscle–joint pain (*n* = 92). The fourth group included those with only joint (*n* = 28) or only muscle (*n* = 67) diagnosis. A single patient could belong to several groups. Females were predominant in all groups (Table 2).

**TABLE 2:** Distribution of women and men in each group.

		Women	Men	Total
Group 1: Muscle diagnosis		126(77.30%)	33(58.92%)	159(72.60%)
Group 2: Joint diagnosis		92(56.44%)	28(50%)	120(54.79%)
Group 3: Comorbid diagnosis		75(46.01%)	17(30.35%)	92(42.00%)
Group 4: Only muscle or joint diagnosis	4a: Only muscle diagnosis	51(31.28%)	16(28.57%)	67(30.59%)
	4b: Only joint diagnosis	17(10.42%)	11(19.64%)	28(12.78%)

### Temporomandibular disorders distribution

The following DC/TMD diagnoses were made in the study group: myalgia, myofascial pain, myofascial pain with referral, tendonitis, disc displacement with reduction, disc displacement without reduction, arthralgia, degenerative joint disease, osteoarthrosis, osteoarthritis, and subluxation.

The most common diagnosis among the studied patients was myalgia. It was observed in 69.86% (*n* = 153) of patients on at least one side, and was dominant in both women (*n* = 121) and men (*n* = 32). The second most frequent diagnosis was disc displacement with reduction in both women (*n* = 60) and men (*n* = 14). The third most common diagnosis was myofascial pain (*n* = 24). A comparison of TMD diagnosis between males and females is presented in Table 3.

**TABLE 3:** Diagnosis distribution in women and men.

	Women	Men	Total
Myalgia	121(74.23%)	32(57.14%)	153(69.86%)
Myofascial pain	21(12.88%)	3(5.35%)	24(10.95%)
Disc displacement with reduction	60(36.80%)	14(25%)	74(33.78%)
Disc displacement without reduction	4(2.45%)	2(3.57%)	6(2.73%)
Arthralgia	43(26.38%)	12(21.42%)	55(25.11%)
Osteoarthritis	8(4.9%)	2(3.57%)	10(4.56%)
Osteoarthrosis	6(3.68%)	0(0%)	6(2.73%)
Subluxation	4(2.45%)	2(3.57%)	6(2.73%)

### Temporomandibular disorders and patient health questionnaire-9

Among the studied patients, 54.80% (*n* = 120) scored more than the cutoff points for depression symptoms, indicating the presence of mild (19.63%, *n* = 43), moderate (14.15%, *n* = 31), severe (13.24%, *n* = 29), and moderately severe depression (7.76%, *n* = 17). No signs of depression were found in (45.20% *n* = 99). The cutoff score of the PHQ-9 questionnaire was exceeded by 58.89% of women and 41.07% of men (Table 4).

**TABLE 4:** Questionnaires scores for women and men.

		Women	Men	Total
PHQ-9^†^	No depression	66(40.49%)	33(58.92%)	99(45.20%)
	Mild depression	31(19.01%)	11(19.64%)	43(19.63%)
	Moderate depression	23(14.11%)	8(14.28%)	31(14.15%)
	Moderately severe depression	16(9.81%)	1(1.78%)	17(7.76%)
	Severe depression	26(15.95%)	3(5.35%)	29(13.24%)
GAD-7^‡^	No anxiety	110(67.48%)	41(73.21%)	151(68.94%)
	Mild anxiety	30(18.40%)	9(16.07%)	39(17.80%)
	Moderate anxiety	18(11.04%)	2(3.57%)	20(9.13%)
	Severe anxiety	5(3.06%)	4(7.14%)	9(4.10%)
GCPS^§^	GCPS 0	7(4.29%)	9(16.07%)	16(7.30%)
	GCPS 1	83(50.92%)	29(51.78%)	112(51.14%)
	GCPS 2	34(20.85%)	7(12.5%)	41(18.72%)
	GCPS 3	24(14.72%)	5(8.92%)	29(13.24%)
	GCPS 4	14(8.58%)	6(10.71%)	20(9.13%)
PSS-10^¶^	Low stress	77(47.23%)	31(55.35%)	108(49.31%)
	Moderate stress	73(44.78%)	21(37.5%)	94(42.92%)
	High perceived stress	13(7.97%)	4(7.14%)	17(7.76%)

^†^Patient Health Questionnaire 9, ^‡^Generalized Anxiety Disorder 7, ^§^Graded Chronic Pain Scale, ^¶^Perceived Stress Scale 10.

The ordered logistic regression showed that patients with muscular TMD scored statistically significantly (*p* = 0.015) higher in the PHQ-9 questionnaire, while the presence of joint diagnosis had no statistically significant effect (*p* = 0.524) (Table 5).

**TABLE 5:** The results of multiple ordered logistic regression of the graded total scores of the GCPS, PSS-10, and PHQ-9 on the presence of muscle diagnosis, the presence of joint diagnosis, age, and sex.

Scale	Predictor	*t* ^†^	*P* ^‡^	OR^§^	Low 95% CI^¶^	High 95% CI^¶^
GCPS	Presence of muscle diagnosis	2.11	0.035	1.93	1.05	3.55
	Presence of joint diagnosis	1.31	0.189	1.40	0.85	2.33
	Age	0.94	0.348	1.01	0.99	1.02
	Sex (males)	–1.34	0.181	0.64	0.34	1.23
PSS-10	Presence of muscle diagnosis	1.74	0.081	1.72	0.93	3.17
	Presence of joint diagnosis	1.08	0.280	1.34	0.79	2.27
	Age	–2.39	0.017	0.98	0.96	1.00
	Sex (males)	–1.05	0.292	0.71	0.38	1.34
PHQ-9	Presence of muscle diagnosis	2.44	0.015	2.18	1.17	4.06
	Presence of joint diagnosis	0.64	0.524	1.19	0.70	2.01
	Age	–1.71	0.087	0.99	0.97	1.00
	Sex (males)	–2.17	0.030	0.50	0.27	0.93
GAD-7	Presence of muscle diagnosis	1.74	0.082	3.09	0.87	10.98
	Presence of joint diagnosis	1.67	0.094	2.13	0.88	5.18
	Age	–1.21	0.225	0.98	0.96	1.01
	Sex (males)	–0.42	0.674	0.80	0.29	2.21

^†^Test value, ^‡^Statistical significance, ^§^Odds ratio, ^¶^The limits of the 95% confidence intervals of OR.

The level of the PHQ-9 index is statistically significantly associated with gender. Among the studied group of women, the values of the PHQ-9 index were higher than in the studied group of men (*p* = 0.030; OR = 0.50) (Table 5).

The relationship between the level of PHQ-9 index and age was marginally significant (*p* = 0.087; OR = 0.99) (Table 5).

Ordered logistic regression also indicates the existence of a relationship between the height of the GCPS index and the value of PHQ-9 (*p* = 0.0005; OR = 1.040) (Table 6).

**TABLE 6:** The results of the three ordinal logistic regressions of GCPS on the total scores of PHQ-9, PSS-10, and GAD-7.

Variable	*t* ^†^	*P* ^‡^	Odds ratio^§^	Low 95% CI^¶^	High 95% CI^¶^
PHQ-9 total score	3.47	0.0015	1.040	1.017	1.063
PSS-10 total score	0.02	1.000	1.017	0.984	1.052
GAD-7 total score	1.45	0.4386	1.041	0.986	1.099

The *p*-values are corrected for controlling the effect of multiple testing with the use of Bonferroni’s correction (multiplication of *p*-value by 3). ^†^Test value, ^‡^Statistical significance, ^§^Odds ratio, ^¶^The limits of the 95% confidence intervals of OR.

### Temporomandibular disorders and generalized anxiety disorder scale-7

Analysis based on GAD-7 questionnaire showed that 68.94% (*n* = 151) of participants did not exceed the first cutoff point for mild anxiety, while 17.80% (*n* = 39) had mild anxiety, 9.13% (*n* = 20) had moderate anxiety, and 4.1% (*n* = 9) had severe anxiety (Table 4). No statistically significant difference was observed between women and men in general (*p* = 0.674); the correlation between the height of the GAD-7 index and age was not found either (*p* = 0.225). The presence of the muscle diagnosis tended to be related to GAD-7 more strongly than the presence of joint diagnosis (OR amounted to 3.09 and 2.13), however both relationships were marginally statistically significant (*p* = 0.082 and *p* = 0.094, respectively) (Table 5).

Ordered logistic regression indicates no statistically significant relationship was found between the height of the GCPS index and the GAD-7 value (Table 6).

### Temporomandibular disorders and graded chronic pain scale

Most of the patients in the study group were rated as grade 1 or 2 based on the GCPS score. Around 51% of women and 51% of men accounted for grade 1 or the group with low disability and low intensity. Around 21% of women and 12.5% of men accounted for grade 2 and had complaints defined as high intensity and low disability. High disability and related moderately or severely limiting complaints were reported by 23.4% of women and 19.5% of men. Only 4.29% of women and 16.07% of men reported no disability (Table 4).

The higher GCPS scores was related to the muscle diagnosis (*p* < 0.035), and no statistically significant relationships with the other diagnoses was found. Age (*p* = 0.348) and gender (*p* = 0.181) do not seem to be correlated with the GCPS level (Table 5).

Patients who presented with a higher degree of disability due to the high intensity of the experienced ailments statistically more frequently showed depressive disorders, with greater severity than patients with lower GCPS scores (*p* < 0.0005; OR = 1.040). However, no correlation was observed between the value of the GCPS index and the value of PSS-10 (*p* = 0.9979; OR = 1.017) and GAD-7 (*p* = 0.1462; OR = 1.041) (Table 6).

### Temporomandibular disorders and perceived stress scale-10

The psychoemotional state of the patients was assessed using the PSS-10 tool, in order to verify whether a relationship existed between pain (a symptom of TMD) and psychosocial status. Depending on the PSS-10 scores, the patients were categorized as groups with low stress, moderate stress, and high perceived stress.

The analysis of stress levels by PSS-10 indicated that 50.68% of the studied population had moderate or high perceived stress. No significant differences were observed in the PSS-10 scores between men and women (*p* = 0.292) Low stress was found in 47.23% of women and 55.35% of men, while moderate stress was found in 44.78 and 37.5%, respectively. High perceived stress was the least frequent and found in only 7.97% of women and 7.14% of men (Table 4). On the other hand, age is a factor that correlates to the height of the PSS-10 index negatively. i.e., the older the age, the lower the scale score (*p* = 0.017).

The higher PSS-10 index values were found in patients with muscle disorder (*p* < 0.081), and the presence of joint diagnosis had no effect (Table 5).

### Perceived stress scale-10 and generalized anxiety disorder scale-7 and patient health questionnaire-9

The use of the Person’s correlation indicates a statistically significant and quite strong inter-correlation between the total scores of the scales: PHQ-9 and PSS-10 (*p* < 0.001), GAD-7 and PSS-10 (*p* < 0.001) as well as PHQ-9 and GAD-7 (*p* < 0.001) (Table 7).

**TABLE 7:** The Pearson’s correlation (coefficients r, *p*-values) between the total scores of PHQ-9, PSS-10, and GAD-7.

Variable	PSS-10 total score	GAD-7 total score
PHQ-9 total score	0.5656 *p* < 0.001	0.6146 *p* < 0.001
PSS-10 total score		0.7257 *p* < 0.001

All the variables were transformed with the use of Box-Cox’s function.

## Discussion

Previous studies have indicated the association between TMD and psychological symptoms such as depression, somatization, and anxiety (Suvinen et al., 2005; Manfredini et al., 2011; Canales et al., 2019). According to some authors, depression and anxiety are risk factors for TMD (Bonjardim et al., 2005; Monteiro et al., 2011) while some reported that a higher percentage of mental health disorders was observed in TMD patients than in the general population (Bonjardim et al., 2005; Monteiro et al., 2011; Yeung et al., 2017). A strong association has been found between the levels of anxiety, depression, and pain (characterized by more severity and greater disability) and a reduction in the quality of life (Bair et al., 2008). Sójka et al. (2019) noted that 44% of students with TMD presented with depression and 74.1% with somatization. However, their study was only based on medical students, who are a specific group burdened with severe chronic stress. Recent research indicates that TMD patients with comorbid neurological conditions show common patterns of signature alterations in brain function. In patients with TMD has been observed changes in the thalamocortical pathway in the functional magnetic resonance study performed. Additionally, a change in functional magnetic resonance recordings was observed in these patients after treatment with occlusal splints. TMD pain in these studies was recognized as a source of peripheral neuropathy causing inflammation in the central nervous system After removing the source of peripheral inflammation, we can see immediate significant changes in brain activity and signal overload during examination by functional magnetic resonance imaging (Barkhordarian et al., 2020). Chen et al. (2022) also indicated neurobiological evidence that the occurrence and development of negative emotions in TMD patients may be related to the dysfunction of dopamine pathway components induced by chronic pain. The abnormal dopaminergic functional connection linking orofacial pain and depression may indicate right treatment of TMD comorbidity with negative emotion by the application of serotonin reuptake inhibitor drugs (Chen et al., 2022). Also studies conducted by Suenaga et al. have shown that TMD pain is most often associated with the thalamus, the primary somatosensory cortex, the insula, and the anterior and mid-cingulate cortices (Suenaga et al., 2016). Investigation conducted by Budd et al. (2022) refers to brain abnormalities seen in patients with TMDs. Their results support that chronic pain can alter the brain’s structure and have profound emotional effects (Budd et al., 2022).

In our study, 54.80% of patients with TMD exceeded the cutoff point for depression. The prevalence of depression among TMD patients is significantly high, as indicated by both the present study and previous reports, and nearly half of the TMD patients exhibit mild, moderate, or severe depression symptoms. Marbach et al. (1998) and Hoffmann et al. (2011) have also highlighted the higher levels of depression and anxiety in TMD patients as compared to normal controls. The results of previous studies are in line with ours, the most important findings of which are as follows: (1) more than half of the studied patients showed higher-than-cutoff scores for the PSS-10 and PHQ-9 questionnaires, the level of the GAD-7 questionnaire is also associated with the presence of TMD, while in the studied group of patients the reported level of anxiety was more often classified as mild and moderate than severe. The collected results show a relationship between TMD and the levels of stress, depression and anxiety.; (2) the levels of depression, stress and perceived anxiety strongly correlated with each other, which indicates the importance of the psychoemotional state of patients among patients with TMD; (3) depression symptoms were higher in females than in males, while the level of anxiety and stress was similar in both groups; (4) the most statistically significant correlation was noted between the group of muscle diagnoses and the level of depression, stress, anxiety and disability; (5) the GCPS scores of the studied TMD patients indicated that most patients described their symptoms as characterized by low intensity or high intensity but low disability; (6) patients with more severe disability, as determined by the GCPS score, had statistically significantly higher PHQ-9 index values, thus representing a group with more severe degree of depression. The results suggested the importance of the psychoemotional status of TMD patients, which should be taken into consideration while making therapeutic decisions for this population.

The study showed that patients with muscular TMD had significantly higher PHQ-9, PSS-10, GAD-7, and GCPS scores. It is worth noticing that woman were significantly more frequently diagnosed with this form of TMD. In our study, there were as many as 74% of women, and muscular TMD was diagnosed in 77.30% of this group. Myalgia was frequently diagnosed in women with higher levels of depression and anxiety, and this association can be attributed to several reasons. The literature indicates that muscle disorders are most commonly observed among TMD patients, and myalgia and myofascial pain are more often found in women than men (Wieckiewicz et al., 2020). Tuuliainen et al. (2015) showed that muscle pain is associated with psychosocial distress. The higher number of women with TMD in our study sample compared to men may be related to hormone fluctuations, biological differences, social position, or higher sensitivity to pain (Abubaker et al., 1993). Thorn et al. (2004) reported that women are more likely to complain about even slight pain in a clinical examination, while men complain only if the pain is severe. Another important factor is the hypothalamic–pituitary–adrenal axis, and it was proven that its hyperactivity increases the level of cortisol, which is found in patients with both depression and facial pain (Korszun, 2002).

The results of our study indicated that women scored higher in PHQ-9 analyses. The cutoff point for the PHQ-9 questionnaire was exceeded by 58.89% of women and 41.07% of men, which indicates that women suffering from TMD are significantly more often diagnosed with depression symptoms. This difference was also noted for PHQ-9 scores, according to which moderate stress and high perceived stress were observed in 52.75% of women and 44.64% of men. The literature also indicates that women scored higher in the assessment of depression and anxiety (Kocalevent et al., 2013). Additionally, the study of Simoen et al showed no differences in PHQ-9 and GAD-7 scores between male TMD patients and the male reference population but strong significant differences in scores between female TMD patients and the female reference population (Simoen et al., 2020).

The findings of our study are contradictory to those found by Lövgren et al. (2018) in their study on dental students. These authors observed no significant difference between dental students with and without TMD in terms of PHQ-9, PSS-10, and GAD-7 scores. Notwithstanding, there are some similarities between their study and the present study; for example, in both studies, myalgia was the most commonly diagnosed disorder. On the other hand, the study by Simoen et al. (2020) showed significant differences in PHQ-9 and GAD-7 scores between the studied group with painful TMD and the general population. Similar results were observed by Manfredini et al. in their study, in which 41% of TMD patients were diagnosed with moderate or severe depression (Manfredini et al., 2010).

In our study, the GCPS scores indicating high disability and related moderately limiting complaints (grade 3) and high disability and severely limiting complaints (grade 4) were found in 13.24 and 9.13% of the respondents, respectively. The ratio of the percentage of TMD patients to individual GCPS scores is very similar to the values described by other authors. In the study of Canales et al. the GCPS scores indicating the most severe degrees of pain-related impairment were 10% for grade 3 and 4.3% for grade 4.33. Similar values for the two most severe GCPS grades were observed by other authors (Ohrbach et al., 2010). Based on our results and a comparison of these with the results of previous works, it can be confirmed that only a small number of TMD patients have severe pain-related impairment and only some suffer from high disability with severely limiting complaints. The majority of patients with TMD describe their complaints as low disability, low intensity (grade 1–51.14% of the studied group) and low disability, high intensity (grade 2–18.72% of the studied group). On the other hand, very few TMD patients will report no disability at all (grade 0).

The studies conducted so far indicate an association between the GCPS index and patients’ response to the treatment protocol, indicating that this index has a significant impact on clinical decisions. Based on our results, it may be suggested that patients with severe disability respond very poorly to treatment, while people with low disability get better even after cognitive behavioral therapy (Manfredini et al., 2013; Canales et al., 2019).

The lack of a control group may be considered as a limitation of this study. As the study was conducted on patients from the outpatient clinic, no healthy people were included. On the other hand, the study provides an insight into the pain intensity, pain-related disability, depression, anxiety, and perceived stress in Polish TMD patients and the interrelationship between TMD and the mentioned psychosocial symptoms. From this point of view, the sample included can be considered is a homogeneous group, which is a strength of the study. Additionally, the study was carried out on a large sample and used validated questionnaires and a well-established protocol (DC/TMD) for clinical examination, which indicate that the collected data are strongly reliable.

Although similar studies have been conducted by other authors, they were related to a different patient population. The authors of these studies have pointed out that due to differences in gender, culture, ethnicity, and healthcare provision, the expression of TMD can vary in patients around the world (Canales et al., 2019). Genetic factors also play an important role, and some of these have been identified as related to myofascial pain in Americans and Europeans (Dunn et al., 2011). Genetics may explain why myofascial pain is more frequently observed among Caucasians compared to Latin Americans (Simoen et al., 2020). Taking into account all the factors that can influence the occurrence of TMD and the psychosocial status of patients, it seems reasonable to conduct research on different populations and compare their results. Therefore, in the future, this type of large-scale study can be conducted on a large group of patients from Eastern Europe.

## Conclusion

This study showed a high prevalence of increased levels of anxiety, depression, perceived stress, pain intensity, and pain-related disability among TMD Polish adults. Furthermore, a strong association was observed between TMD and psychological symptoms such as depression, anxiety, and stress in the studied group. This suggests that Polish adult patients with TMD should be screened for depression, stress, and anxiety using tools such as PHQ-9, PSS-10, and GAD-7 during clinical testing. The psychosocial status of Polish TMD patients is an important factor for selecting proper diagnosis and management protocol as it can have a significant impact on the pain intensity, pain-related disability and the patient’s response to TMD treatment. The questionnaires used in the study may allow clinicians to identify patients with a high level of anxiety, stress, or depression, and also to determine the need for guidance and further referral of adult patients to comprehensive diagnostics, and appropriate management or treatment based on an interdisciplinary approach.

## Data availability statement

The raw data supporting the conclusions of this article will be made available by the authors, without undue reservation.

## Ethics statement

The studies involving human participants were reviewed and approved by the Bioethical Committee of the Wroclaw Medical University (Approval No. KB-165/2021). The patients/participants provided their written informed consent to participate in this study.

## Author contributions

MW contributed to study conceptualization. MW and SO recruited the participants. MW, SO, and PS were involved in the management of participants. PS curated the data. NG and KK performed data analysis. MW, PS, SO, AP, and KK prepared the manuscript. OW-A, AE-P, and AJ edited the final version of the manuscript. All authors have read and approved the final manuscript.

## Footnotes

1
https://ubwp.buffalo.edu/rdc-tmdinternational/

2
https://ubwp.buffalo.edu/rdc-tmdinternational/tmd-assessmentdiagnosis/dc-tmd/

3
https://www.mededportal.org/publication/9946/
